# Identifying candidate structured RNAs in CRISPR operons

**DOI:** 10.1080/15476286.2022.2067714

**Published:** 2022-05-01

**Authors:** Brayon J. Fremin, Nikos C. Kyrpides

**Affiliations:** a Department of Energy, Joint Genome Institute, Berkeley, CA, USA; b Environmental Genomics and Systems Biology Division, Lawrence Berkeley National Laboratory, Berkeley, CA, USA

**Keywords:** CRISPR, structured RNA, comparative genomics

## Abstract

Noncoding RNAs with secondary structures play important roles in CRISPR-Cas systems. Many of these structures likely remain undiscovered. We used a large-scale comparative genomics approach to predict 156 novel candidate structured RNAs from 36,111 CRISPR-Cas systems. A number of these were found to overlap with coding genes, including palindromic candidates that overlapped with a variety of Cas genes in type I and III systems. Among these 156 candidates, we identified 46 new models of CRISPR direct repeats and 1 tracrRNA. This tracrRNA model occasionally overlapped with predicted *cas9* coding regions, emphasizing the importance of expanding our search windows for novel structure RNAs in coding regions. We also demonstrated that the antirepeat sequence in this tracrRNA model can be used to accurately assign thousands of predicted CRISPR arrays to type II-C systems. This study highlights the importance of unbiased identification of candidate structured RNAs across CRISPR-Cas systems.

## Introduction

CRISPR-Cas (clustered regularly interspaced short palindromic repeats-CRISPR associated) systems are utilized by bacteria and archaea to protect themselves against infectious agents. These systems use RNA-guided nucleases to target and cut specific sequences of double-stranded DNA [1]. Noncoding RNAs play central roles in CRISPR-Cas systems. CRISPR RNAs (crRNAs) are noncoding RNAs that are transcribed and processed by enzymes encoded in the CRISPR sequence array, which contains direct repeats separated by spacers. The crRNAs guide Cas nucleases to their target DNA sequences and are found in all known CRISPR-Cas systems [2]. Transactivating CRISPR RNAs (tracrRNAs) are noncoding RNAs encoded by type II and some type V CRISPR-Cas systems that aid in maturation of crRNAs and DNA cleavage by CRISPR-Cas9 [3,4]. Short complementarity untranslated RNAs (scoutRNAs) are recently discovered noncoding RNAs that assemble with Cas12c/d and crRNA to function as a DNA-targeting complex [5]. Thus, discovery of additional noncoding RNAs associated with CRISPR-Cas systems will likely be important for understanding the mechanisms and adaptation of CRISPR-Cas systems.

All of these noncoding RNAs associated with CRISPRs have been shown or predicted to form secondary structures [5,6]. Currently, there are 64 families of direct repeats and one family of tracrRNAs in Rfam [7]. More diversity exists within crRNAs and tracrRNAs that existing models do not capture. It is likely that other noncoding RNAs exist that play essential roles in CRISPR-Cas systems that have yet to be discovered, and these noncoding RNAs may also form secondary structures [5]. Additionally, there likely exists substantial diversity in structures within crRNAs and tracrRNAs that have yet to be identified. Building additional models would be beneficial both to characterize the structures as well as better search for them in genomes. There is also precedence for regulatory RNAs being embedded in bacterial coding regions [8–10]. Because most of the focus is on intergenic regions, regulatory RNAs that overlap genes tend to be overlooked. However, this is an important consideration from a genetic engineering perspective; perhaps upon codon optimization of a Cas gene, for example, the structure and function of an essential overlapping noncoding RNA is disrupted. This perspective motivated us to predict candidate structured RNAs and include coding regions in our analyses. Though comparative genomics approaches have yet to be applied to Cas operons and CRISPRs at large-scale, it has previously been a useful approach to predict candidate structured RNAs in microbiomes [11–13].

In this work, we used a comparative genomics approach to predict candidate structured RNAs across 15,144 Cas operons, 21,141 associated CRISPRs, and 20,967 orphan Cas operons from diverse ecosystems. This approach involved clustering conserved regions within CRISPRs and Cas operons, predicting possible structures, and assessing possible structures for evidence of covariation, which would indicate evolutionary constraint to preserve the structure. Overall, our pipeline predicted 156 novel candidate structured RNAs, including 1 tracrRNA. Of these 156 candidate structured RNAs, 99 overlapped coding regions, 46 were novel direct repeats,and 11 were in intergenic regions. In addition to substantially expanding upon the diversity of known RNA structures, this approach predicted palindromic candidates overlapping Cas genes and novel candidates in intergenic regions across diverse CRISPR-Cas systems. Additionally, we showed that the antirepeat region of our novel tracrRNA model can be used to accurately assign 4,661 CRISPR arrays to type II-C systems based on homology between the array repeats and tracrRNA antirepeats.

## Results

CRISPRCasTyper [14] was used to predict CRISPRs and Cas operons in ~25 million contigs (>3kb) from 29,521 publicly available and published metagenomics assemblies in IMG/M [15–38]. We identified 15,144 Cas operons associated with 21,141 nearby CRISPR arrays and an additional 20,967 orphan Cas operons, which were all used to predict candidate structured RNAs. To avoid false positives, we did not consider isolated CRISPR arrays or putative Cas operons in these analyses.

**Figure 1.**
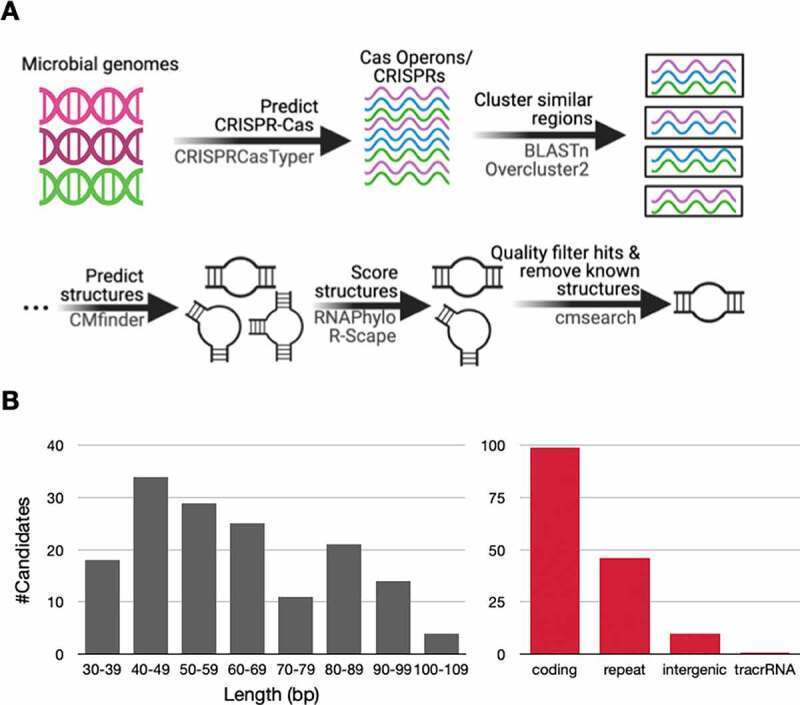
Prediction of candidate structured RNAs.

The first step for predicting candidate structured RNAs was to identify conserved regions along these Cas operons and CRISPRs. Conserved regions were identified using all versus all BLASTn [39], querying all Cas operons and associated CRISPR arrays against themselves (Fig. 1A). We filtered these blast results to exclude 100% identity matches, hits that span less than 30 bases in length, and hits with bit scores below 20. We set these filters because we ultimately wanted to align homologous regions that contained nucleotide differences to assess covariation. We clustered homologous regions into 11,546 clusters using overcluster2 with default settings. The next step was predicting structures. Using CMfinder [40,41], we generated motifs for 7,173 of these clusters. Using RNAphylo, a tool using phylogenetic models to score alignments, we found that 1,741 clusters contained motifs with an RNAphylo p score of 10 or greater. Additionally, we found that 717 of these contained at least one significant covarying base using R-scape [42]. Using cmsearch [40], we determined which of these alignments significantly (*E* value < 1 × 10^−6^) hit at least three regions near Cas operons and associated CRISPRs. We removed duplicates that hit any of the same regions as another candidate structured RNA, selecting the longest candidate. This resulted in a set of 159 candidate structured RNAs. Three of these 159 candidate structures were CRISPR direct repeats already found in Rfam [7]. In fact, Rfam contains 64 models of CRISPR direct repeats and 34 of these were identified in the 36,111 CRISPR-Cas systems we searched. 31 of these models did not meet the stringent phylogenetic and covariation thresholds we set, suggesting a high false negative rate using our approach.

Using this comparative genomics approach resulted in a finalized set of 156 novel candidate structured RNAs. These 156 novel structural RNAs were identified in 6,509 instances from 36,111 systems searched (Fig. 1A, File S1, File S2). The average length was 62 bases (range 32 to 109 bases) (Table S1, Fig. 1B). We taxonomically classified 150 candidates to Bacteria and 6 to Archaea. Furthermore, 91 candidates were identified in *Proteobacteria*, 26 in *Firmicutes*, and 12 in *Bacteroidetes* (Table S1). These candidates also belonged to a diversity of subtypes. For example, we identified 34, 3, 15, 1, 4, and 2 candidates in I-C, II-A, III-A, IV-A1, V-A, and VI-B1 systems, respectively (Table S1). We binned these candidates into four categories: candidates that overlapped coding regions, candidates that modelled CRISPR direct repeats, candidates found exclusively in other intergenic regions, and candidates likely to be tracrRNAs (Table S1, Fig. 1B). Below we highlight interesting candidate structured RNAs for each of these categories.

**Figure 2.**
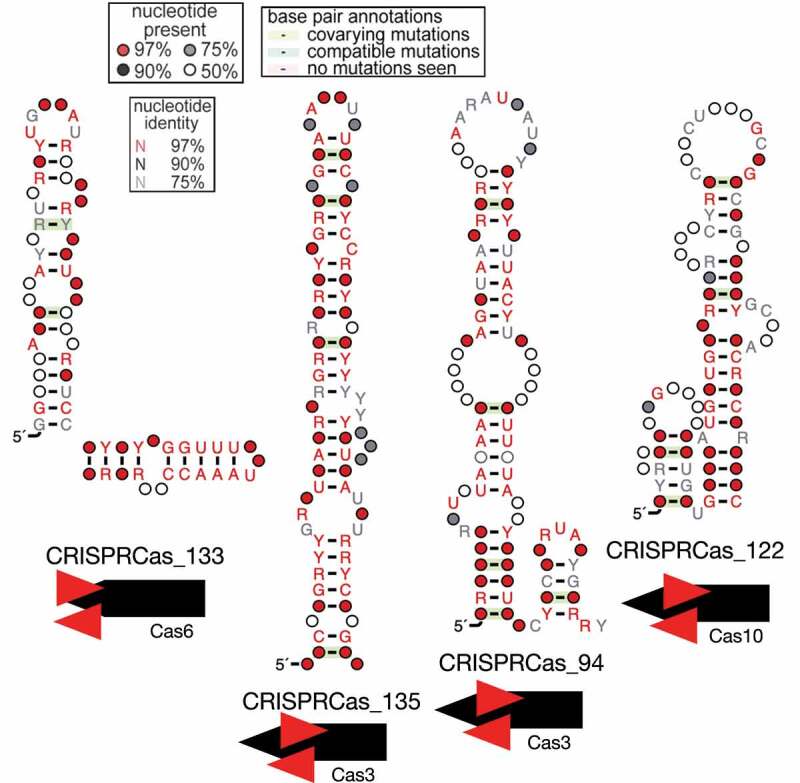
Palindromic candidates overlapping Cas genes.

Upon first inspecting candidates that overlapped coding regions, we identified palindromic candidate structured RNAs that overlapped *cas* genes. CRISPRCas_133 was a palindromic candidate structured RNA identified in 15 CRISPR-Cas type I-B systems that overlapped *cas6* near the end of the gene (typically overlapping the stop codon). It was predominately classified to *Bacteroidetes* and was found twice in the human digestive system, five times in freshwater, and four times in endoliths (Fig. 2, Table S1). CRISPRCas_135 was found in 6 CRISPR-Cas type I-F systems and overlapped *cas3*. It was predominately classified to *Firmicutes* and was found in diverse ecosystems, including the human digestive system and hydrothermal vents. CRISPRCas_94 was found in 13 CRISPR-Cas type I-B systems and also overlapped *cas3*. It was classified as *Firmicutes* and found in clay. Three other palindromic candidate structured RNAs were also predicted to overlap *cas3*; these candidates also occurred in a similar relative position along the gene. CRISPRCas_122 was found in 19 CRISPR-Cas type III-B systems and overlapped *cas10*. It was classified as *Proteobacteria* and has so far only been found in bioreactor samples (Fig. 2). Two other palindromic candidates also overlapped *cas10* in a similar relative position. In addition to these examples, two palindromic candidate structured RNAs overlapped *cas7*, both near the middle of the gene. One palindromic candidate overlapped *cas1* closer to the 3’ end of the gene. One palindromic candidate overlapped *cas8* closer to the 5’ end of the gene (Table S1). One palindromic candidate overlapped *cas4* closer to the 5’ end of the gene. Overall, there seemed to be an intriguing pattern of palindromic candidate structured RNAs overlapping a wide variety of *cas* genes.

**Figure 3.**
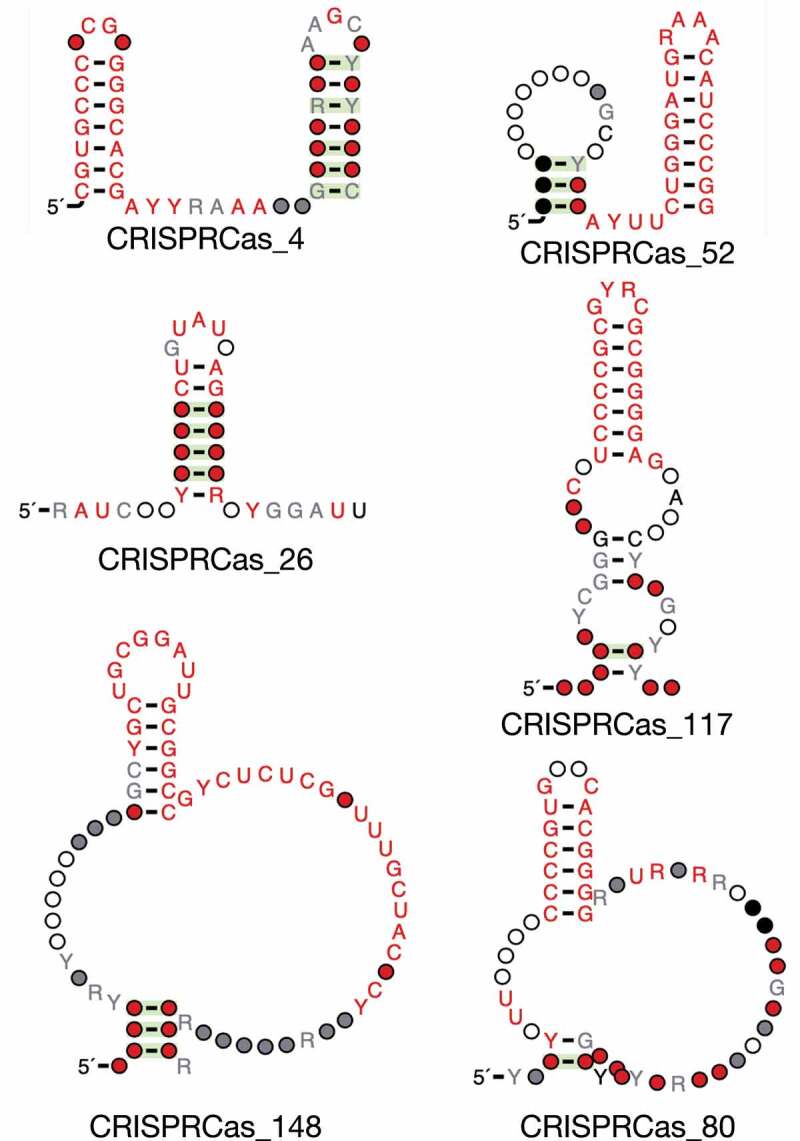
CRISPR direct repeat predictions.

Of the 156 candidate structured RNAs, 46 (29%) were predicted to be direct repeats in CRISPR arrays. These candidates all overlapped CRISPR arrays predicted by CRISPRCasTyper, specifically repeat regions, and occurred across multiple types of arrays. These repeats were typically specific to subtypes of CRISPR-Cas systems. For example, CRISPRCas_4 and CRISPR_52 were direct repeats associated with I-C and I-G systems, respectively (Fig. 3). CRISPR_148 was a direct repeat in II-C systems. CRISPR_26 was a direct repeat in III-A systems. CRISPRCas_80 was a direct repeat in IV-A1 systems, and CRISPRCas_117 was a direct repeat in VI-B1 systems (Fig. 3). Rfam currently contains 64 families of direct repeats. This work further expands this set to 110 distinct models.

**Figure 4.**
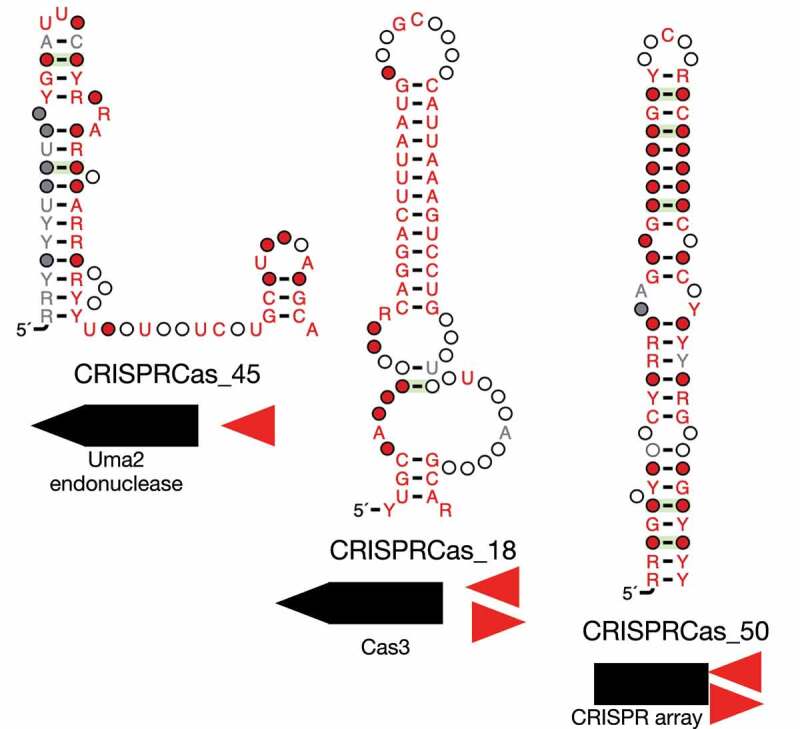
Candidate structured RNAs in intergenic regions.

Upon inspection of predictions found exclusively in intergenic regions, we highlight three interesting candidate structured RNAs. CRISPRCas_45 was located approximately 400 bases upstream of Uma2 family endonucleases found in type V-A systems (Fig. 4, Table S1). It was found entirely in the human digestive system in *Eubacterium* species. CRISPRCas_18 was a palindromic candidate found both in *Proteobacteria* and *Firmicutes* and located approximately 300 bases upstream of *cas3* in type I-C systems. It was found in wastewater and bioreactor samples. CRISPRCas_50 was also a palindromic candidate and was found directly adjacent to CRISPR arrays (typically ~50 bases away) in type I-G systems. It was located in endoliths samples and found both in *Proteobacteria* and *Actinobacteria* (Fig. 4).

**Figure 5.**
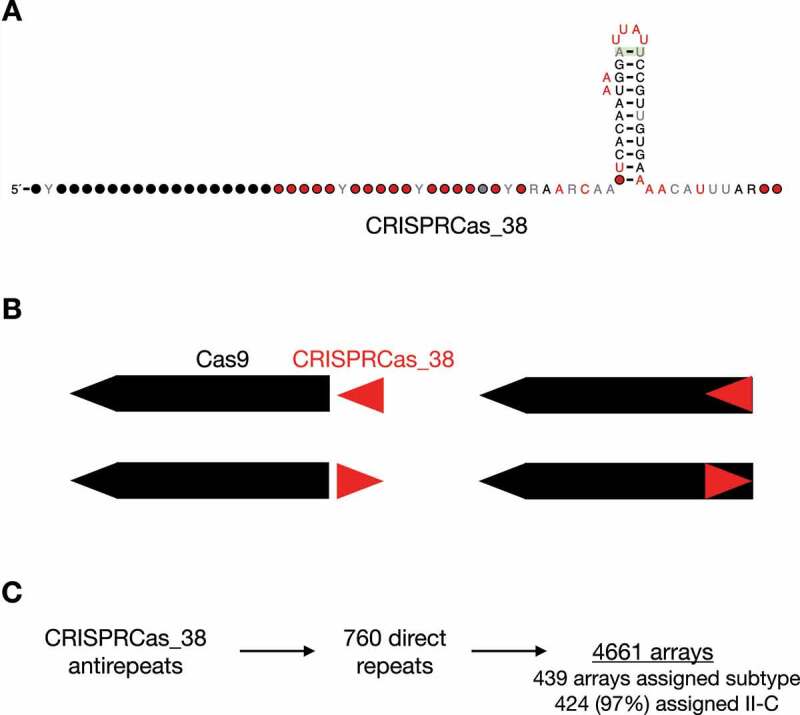
tracrRNA prediction.

One candidate structured RNA, CRISPRCas_38, was likely a novel tracrRNA model (Fig. 5A). CRISPRCas_38 was predominantly found in *Bacteroidetes* (578/599 instances) and was broadly distributed across ecosystems, located 126 times in environmental, 340 times in host-associated, and 133 times in engineered ecosystems. It was typically found in the intergenic region within 100 bases upstream of *cas9* (Fig. 5B). However, in 50 of the 599 genomic positions in which it was identified, it partially or entirely overlapped the start of *cas9*. One likely explanation is that the start site of *cas9* has been occasionally misassigned by Prodigal. Nonetheless, this suggests that it is important to search for structured RNAs even across predicted coding regions. CRISPRCas_38 was found 599 times exclusively in type II-C systems. The antirepeat region of CRISPRCas_38 was homologous to 760 unique repeat regions identified by CRISPRCasTyper (BLASTn e-value < 0.05). These 760 regions were found in 4,661 CRISPR arrays. Interestingly, only 439 of these arrays were assigned a subtype by CRISPRCasTyper and 424 (97%) of those were assigned type II-C (Fig. 5C). For the 15 assigned a different subtype, the subtype probabilities assigned by CRISPRCasTyper ranged from 0.23 to 0.879. There were 310 CRISPR arrays with subtype probabilities greater than 0.9, and all were assigned to type II-C. Using the antirepeat region of this novel tracrRNA model, this suggests we can accurately classify thousands of these CRISPR arrays as type II-C based on their repeat sequence even if the repeat is not near type II-C Cas operons.

## Discussion

As evident by the recent discovery of scoutRNAs [5], it is likely that other key noncoding RNAs that form secondary structures in CRISPR-Cas systems exist but have not been discovered. As metagenomics data becomes increasingly more available and improved tools are developed to predict CRISPR-Cas systems, it becomes possible to mine CRISPR-Cas systems at large-scale to predict novel candidate structured RNAs. In this work, we used publicly available, published datasets available through IMG/MER from diverse microbes and ecosystems and the recently developed tool, CRISPRCasTyper, to predict tens of thousands of Cas operons and CRISPRs and mine them to identify 156 candidate structured RNAs.

There are several limitations to our approach, many of which are similar to limitations of previous approaches [11–13]. First, very few of these candidates were found in metatranscriptomics-associated samples, and we were unable to quantify expression of these candidate structured RNAs. We also did not validate RNA structures experimentally, which would involve using methods like SHAPE-Seq or FragSeq [43–45]. Certain regions of the predicted candidate structures, especially those with less covariation evidence, may not be accurately depicted by this analysis. Second, it was difficult to accurately calculate a false-positive rate for our analyses. Thus, these predictions should be treated as candidates until further followup is performed. Third, we expected a high false-negative rate in these predictions given the scoring metrics and covariation requirements set. For example, we were unlikely to predict rare or highly conserved candidates, which would be difficult to assess for covariation. For example, 34 of the 64 direct repeats present in Rfam were identified in the CRISPR arrays we searched; however, we only rebuild models for 3 of these with our pipeline. There was not enough sequence divergence within our set to build models with significant covariation for the remaining 31 Rfam structures. In fact, most direct repeat models in Rfam do not display significant covariation and would not be retained by our pipeline. Fourth, we could not assign functions to these candidates.

Overall, we provided 156 candidate structured RNAs predicted from Cas operons and CRISPRs. Though follow up work is necessary to validate these candidates, we confidently identified 46 new direct repeats and 1 tracrRNA. We show that the discovery of this tracrRNA model can be useful to improve assignment of CRISPR arrays to type II systems. We also propose some especially interesting candidates, including palindromic candidate structured RNAs that overlap *cas1, cas3, cas4, cas6, cas7, cas8*, and *cas10*. Perhaps these candidates play roles in crRNA maturation or regulation of gene expression, for example, though more work is needed to assign such functions. Nonetheless, if any of these candidates play essential roles in these CRISPR-Cas systems, they will require consideration upon codon optimization of Cas and associated genes and meeting the system requirements from a genetic engineering perspective. We anticipate this resource will prompt experimental characterization, improve searchability of structured RNAs in CRISPR-Cas systems, and may have broader implications in adapting diverse CRISPR-Cas systems for genetic engineering purposes.

## Methods

### Data download and processing

All publicly available assembled metagenomic data with associated publications in IMG/MER were downloaded. We only considered contigs greater than 3 kb for analysis. This resulted in 25,658,797 contigs containing a total of 212,328,312,212 bases. We predicted Cas operons and CRISPRs along these contigs with CRISPRCasTyper version 1.6.1 [14] using default settings. Only regions predicted to be Cas operons or CRISPRs associated with Cas operons were considered for further analysis. We extended these Cas operon and CRISPR regions by 500 bp upstream and downstream using BEDTools slop [46] to capture regions between Cas operons and CRISPR arrays as well as upstream or downstream regions that may be within the operons. These regions were merged together with BEDTools merge, and the sequences corresponding to these regions were isolated using BEDTools getfasta. This resulted in 38,202 regions containing 285,229,248 bases, which we used to search for candidate structured RNAs.

### Predicting candidate structured RNAs

We used BLASTn 2.5.0 +[39] with default settings to identify homologous regions within these Cas operons and CRISPRs. We retained matches with nucleotide differences, alignment lengths of at least 30, and bit scores of at least 20. Regions were clustered together using a single-linkage clustering algorithm, overcluster2, with default settings (Weinberg, Z., unpublished open-source software, available at http://weinberg-overcluster2.sourceforge.io), resulting in 11,546 clusters. We extracted sequences for these clusters using BEDTools getfasta. These clusters were structurally aligned using CMfinder version 0.4.1 [40], resulting in alignments for 7,173 cluster^s^. We scored motifs using RNAPhylo, requiring a p-score of at least 10, filtering to 1,741 clusters. Motifs were also scored for significant covariation (*E* < 0.05) using R-scape [42] with default settings, further filtering to 717 clusters. Using motifs that passed above thresholds, we performed cmsearch [40] of candidate motifs against Cas operons and CRISPRs, retaining those models that uniquely and significantly (*E* value < 1 × 10^−6^) hit at least three unique regions across the regions. This ensured that the models were searchable and unique. Only 159 alignment files were retained from these analyses. We performed cmsearch [40] of Rfam 14.7 [7] against Cas operons and CRISPRs, considering those that meet the GA cut-off. Using BEDTools [46] intersect, we discarded the candidate structured RNAs that overlapped with any regions that were also predicted to be structures in Rfam, resulting in a total of 156 new candidate structured RNAs. RNA structure renderings were drawn using R2R [47]. The highlighted covariation in the renderings indicate bases with significant covariation predicted by R-scape [42]. Additionally, we used R-scape with – fold option to improve covariation among these alignments [48]. To determine if the candidate structured RNAs were found in coding or noncoding regions, we assessed which RNAs overlapped genes predicted by Prodigal [49]. We annotated genes using BLASTp to the nr database. Taxonomy of each contig was assigned using One Codex [50].

### Identifying repeats with homology to tracrRNA antirepeats

Using BLASTp, we queried all predicted repeats identified with CRISPRCasTyper against all the tested Cas operons and CRISPRs. We removed significant hits (e value < 0.05) to CRISPR arrays (self matches) using BEDTools intersect. We then used BEDTools intersect to determine if any candidate structured RNAs were identified in regions homologous to the direct repeats. The only overlap identified was to the candidate structured RNA CRISPRCas_38, which was found in regions that were homologous to 760 distinct direct repeat sequences that could be traced back to 4,661 CRISPR arrays.

## Supplementary Material

Supplemental Material

## Data Availability

This article generated no new sequencing data and included all results of analyses performed. Models are provided as supplemental files. Code used can be found on github: https://github.com/bfremin-lbl/Candidate-Structures-CRISPR-Operons.
