# Supplementary material for: Identifying candidate structured RNAs in CRISPR operons
Source: RNA Biol. 2022 May 1;19(1):678–85. doi: 10.1080/15476286.2022.2067714 (PMC9067536; doi:10.1080/15476286.2022.2067714)

CRISPRCas_100_1

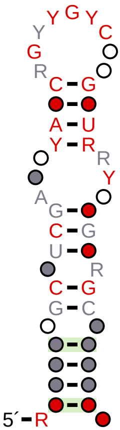

CRISPRCas_101_1

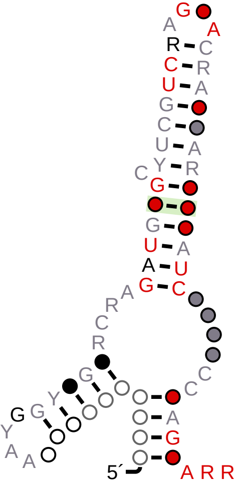

CRISPRCas_10_1

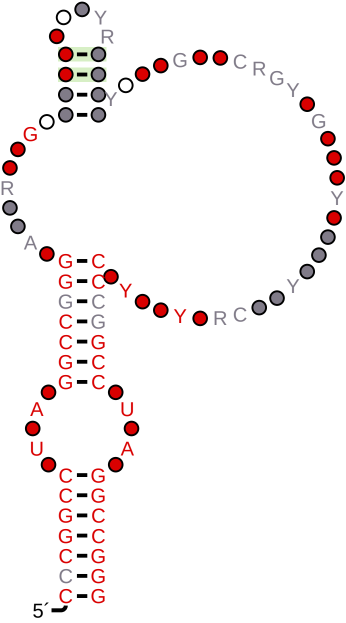

CRISPRCas_102_1

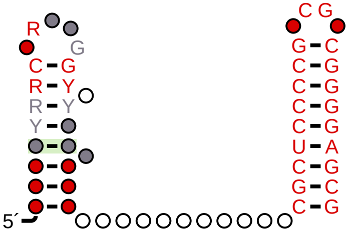

CRISPRCas_103_1

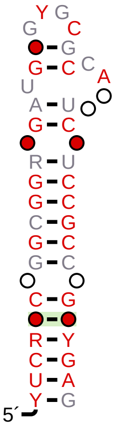

CRISPRCas_104_1

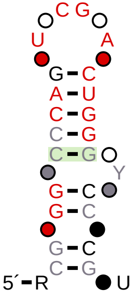

CRISPRCas_105_1

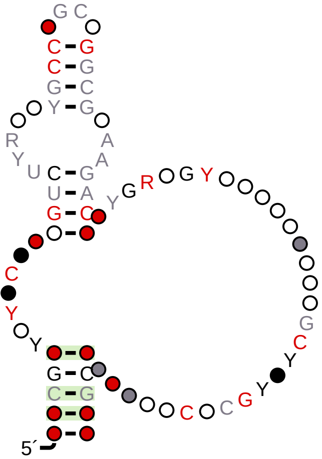

CRISPRCas_106_1

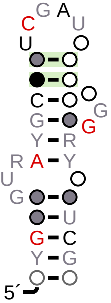

CRISPRCas_107_1

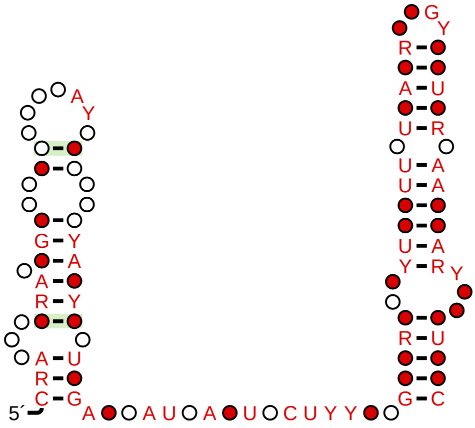

CRISPRCas_108_1

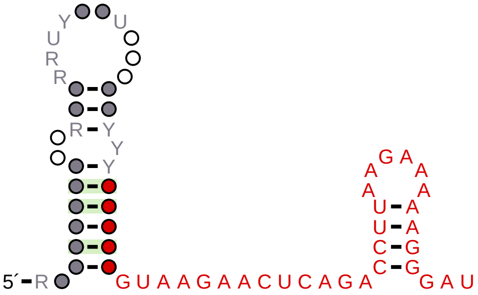

CRISPRCas_109_1

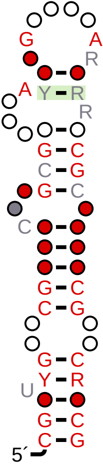

CRISPRCas_110_1

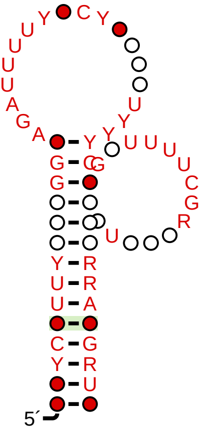

CRISPRCas_111_1

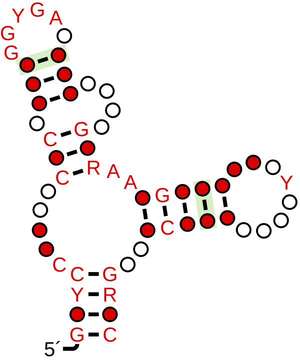

CRISPRCas_11_1

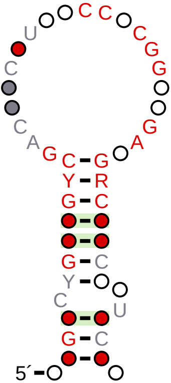

CRISPRCas_112_1

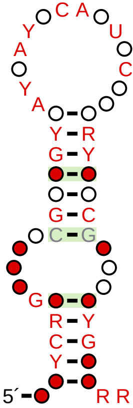

CRISPRCas_113_1

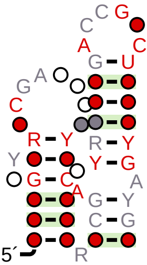

CRISPRCas_114_1

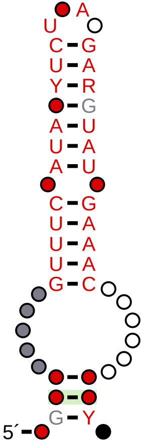

CRISPRCas_115_1

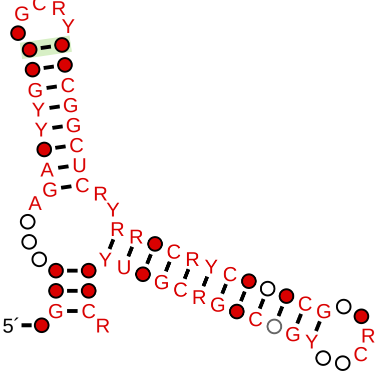

CRISPRCas_116_1

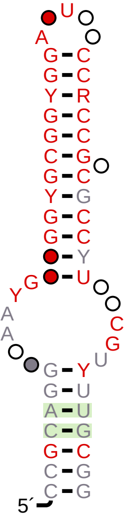

CRISPRCas_117_1

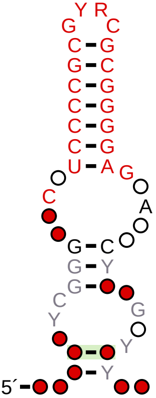

CRISPRCas_118_1

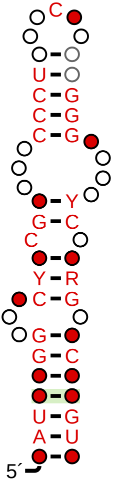

CRISPRCas_119_1

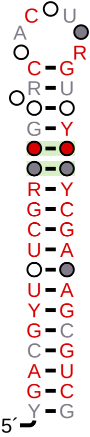

CRISPRCas_1_1

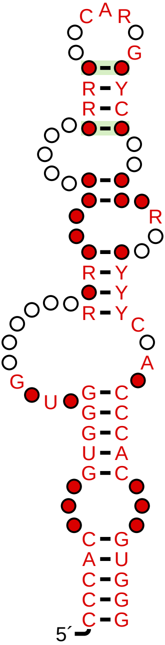

CRISPRCas_120_1

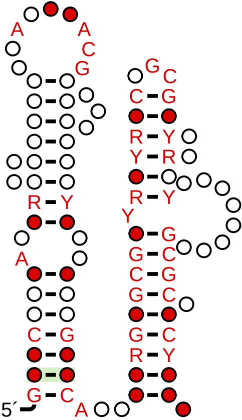

CRISPRCas_121_1

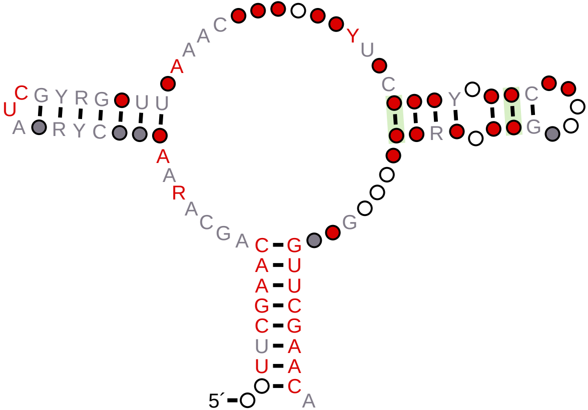

CRISPRCas_12_1

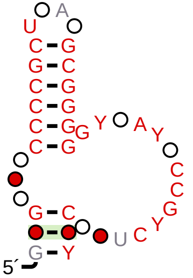

CRISPRCas_122_1

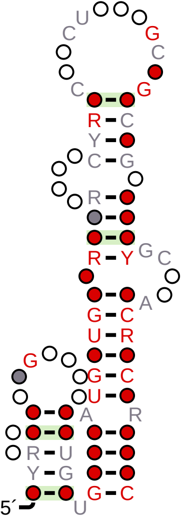

CRISPRCas_123_1

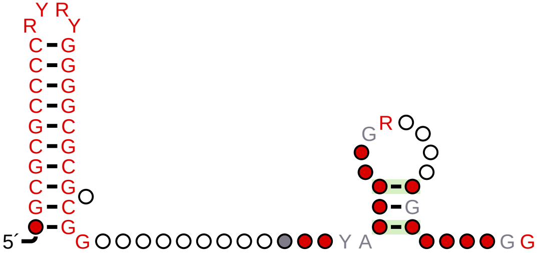

CRISPRCas_124_1

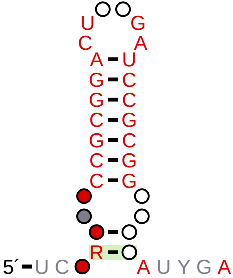

CRISPRCas_125_1

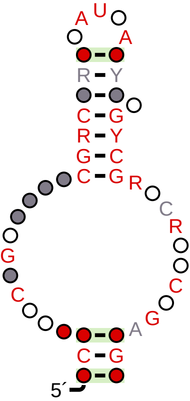

CRISPRCas_126_1

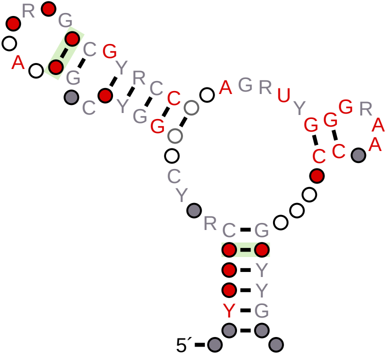

CRISPRCas_127_1

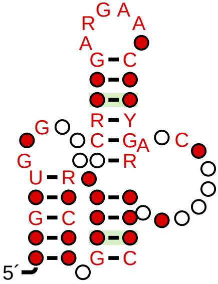

CRISPRCas_128_1

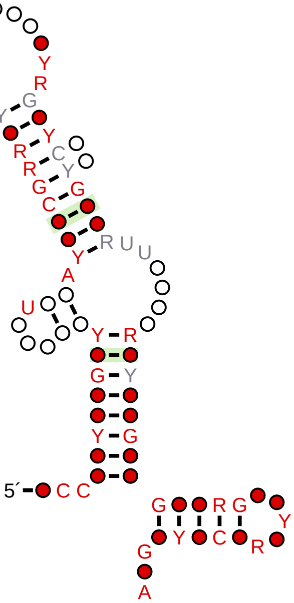

CRISPRCas_129_1

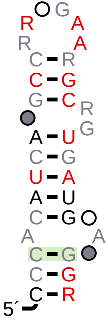

CRISPRCas_130_1

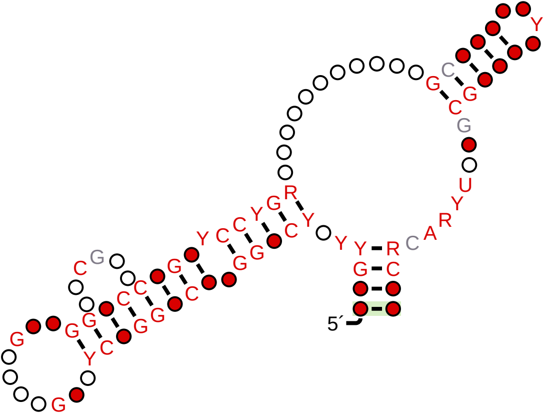

CRISPRCas_131_1

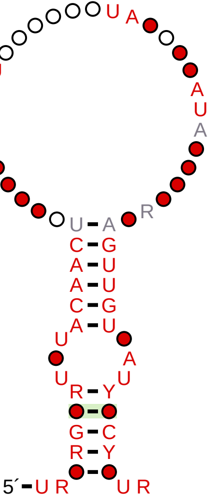

CRISPRCas_13_1

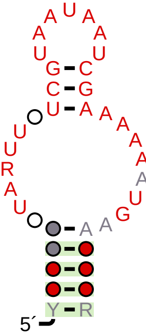

CRISPRCas_132_1

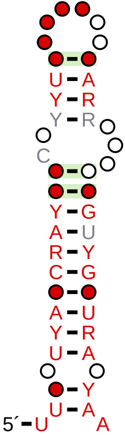

CRISPRCas_133_1

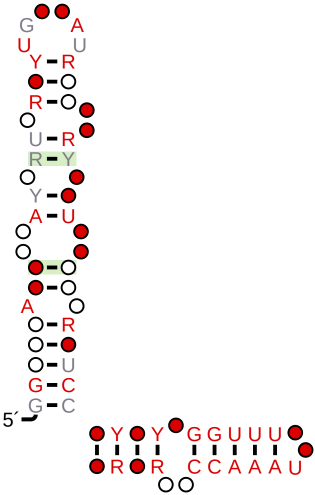

CRISPRCas_134_1

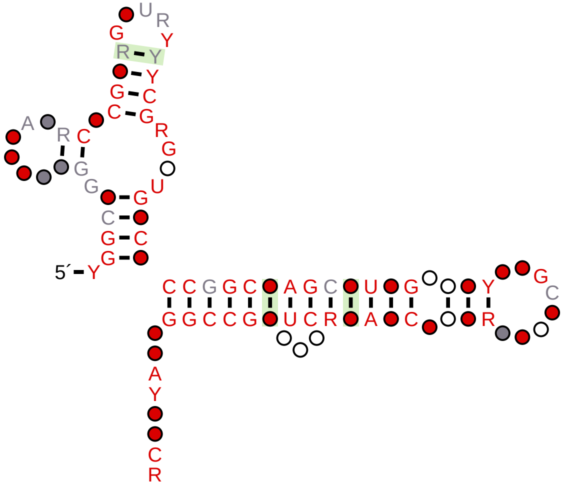

CRISPRCas_135_1

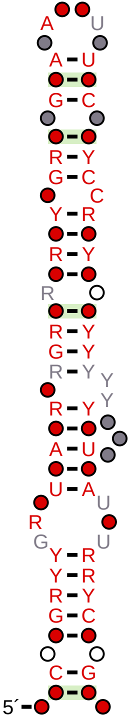

CRISPRCas_136_1

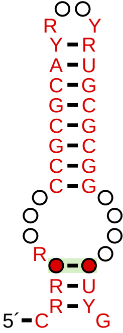

CRISPRCas_137_1

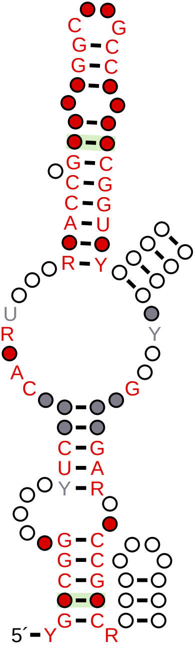

CRISPRCas_138_1

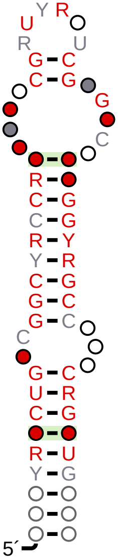

CRISPRCas_139_1

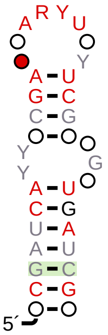

CRISPRCas_140_1

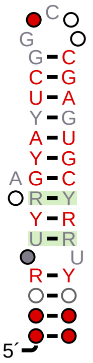

CRISPRCas_141_1

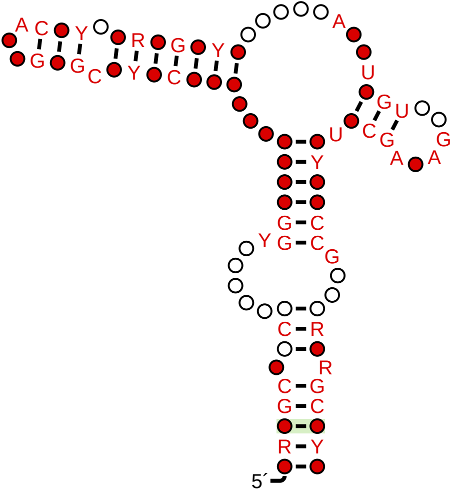

CRISPRCas_14_1

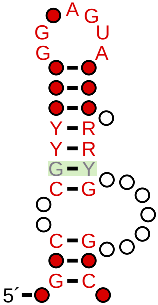

CRISPRCas_142_1

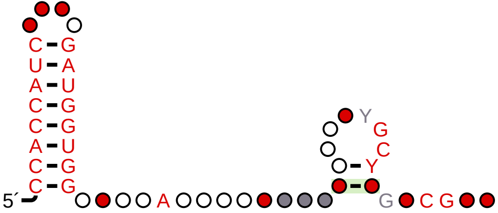

CRISPRCas_143_1

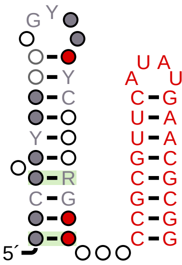

CRISPRCas_144_1

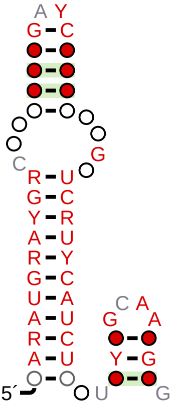

CRISPRCas_145_1

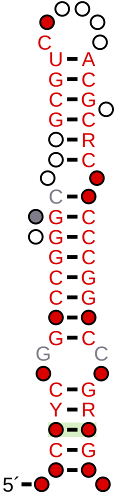

CRISPRCas_146_1

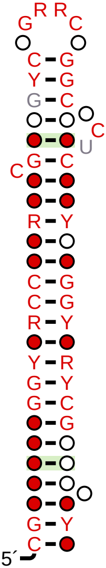

CRISPRCas_147_1

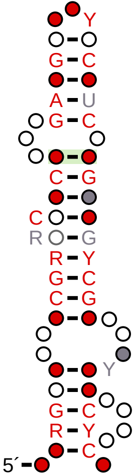

CRISPRCas_148_1

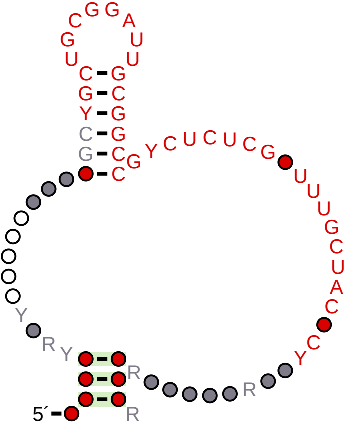

CRISPRCas_149_1

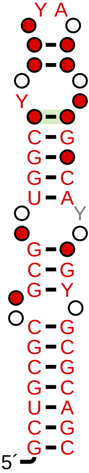

CRISPRCas_150_1

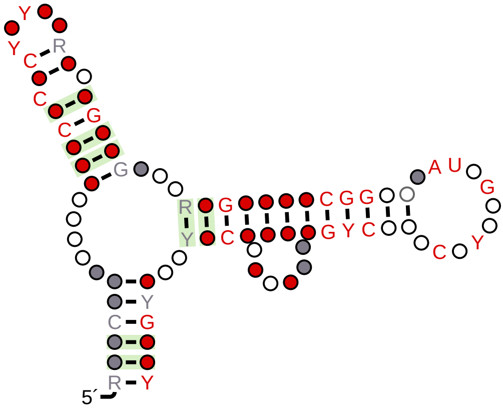

CRISPRCas_151_1

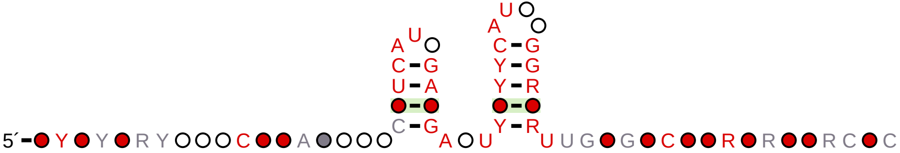

CRISPRCas_15_1

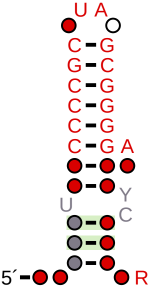

CRISPRCas_152_1

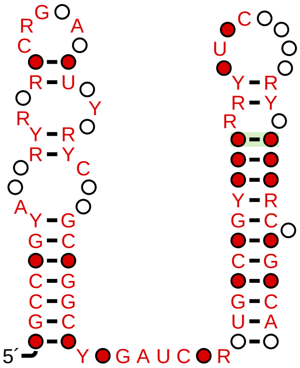

CRISPRCas_153_1

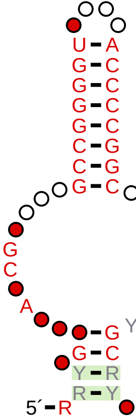

CRISPRCas_154_1

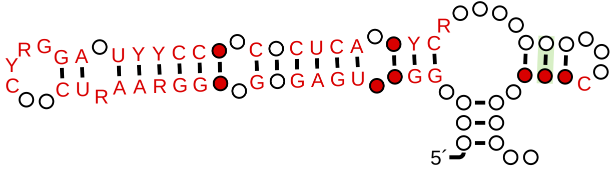

CRISPRCas_155_1

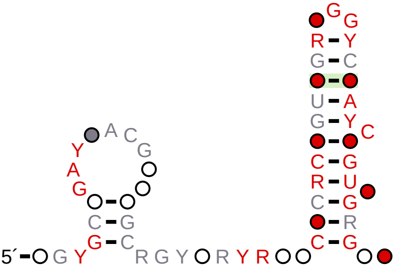

CRISPRCas_156_1

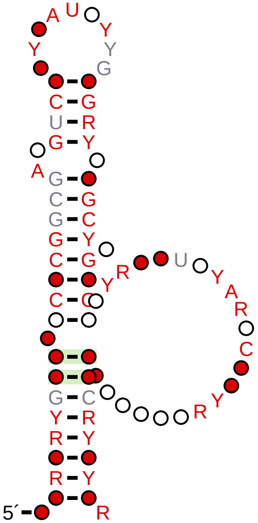

CRISPRCas_16_1

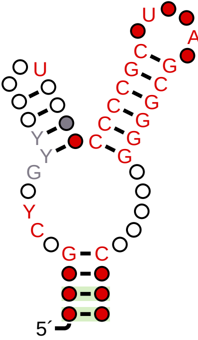

CRISPRCas_17_1

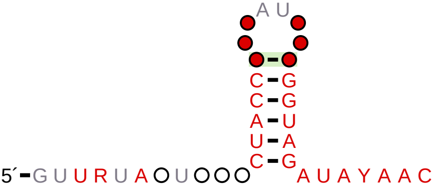

CRISPRCas_18_1

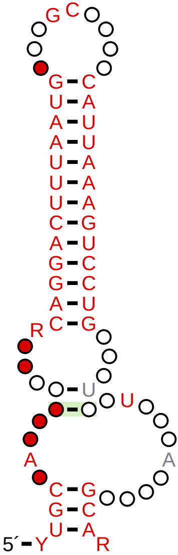

CRISPRCas_19_1

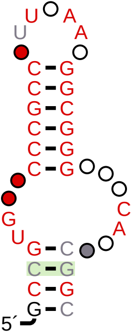

CRISPRCas_20_1

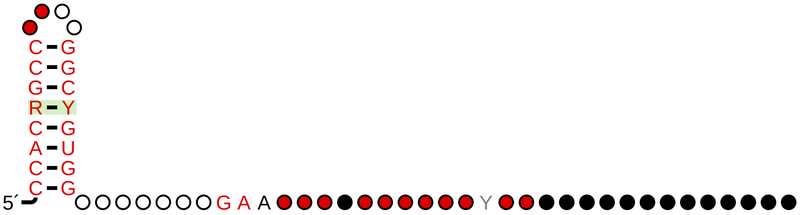

CRISPRCas_21_1

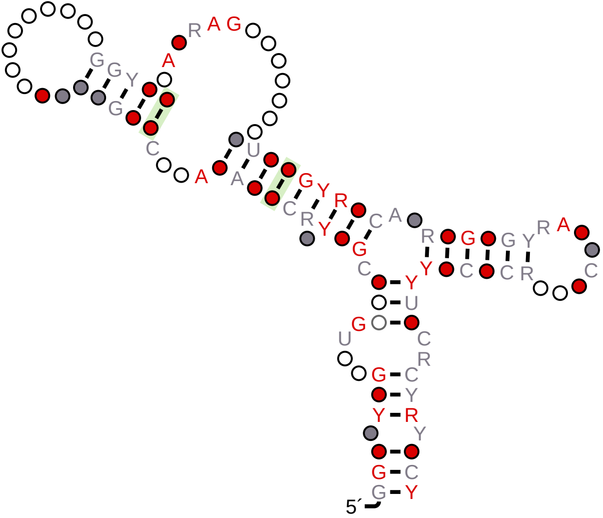

CRISPRCas_2_1

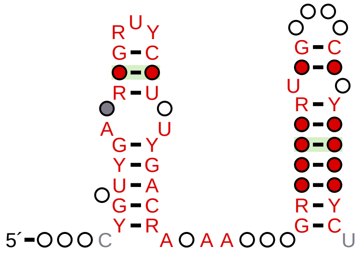

CRISPRCas_22_1

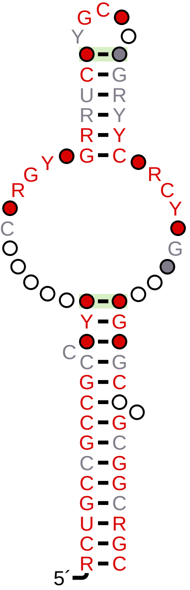

CRISPRCas_23_1

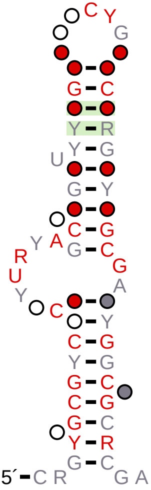

CRISPRCas_24_1

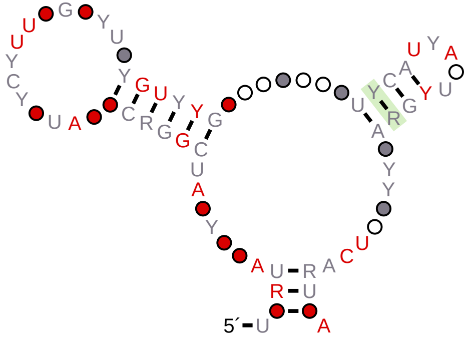

CRISPRCas_25_1

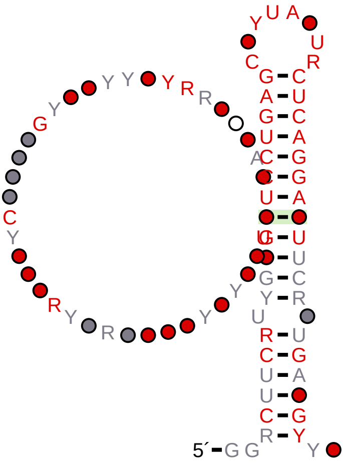

CRISPRCas_26_1

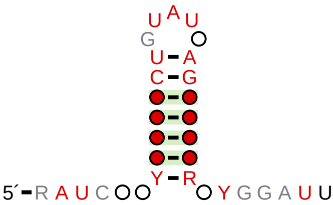

CRISPRCas_27_1

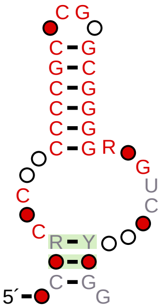

CRISPRCas_28_1

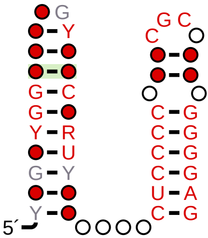

CRISPRCas_29_1

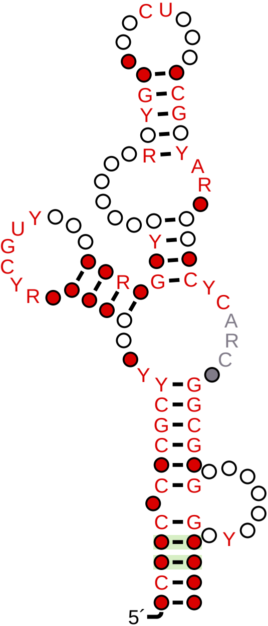

CRISPRCas_30_1

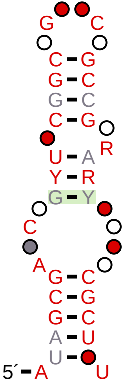

CRISPRCas_31_1

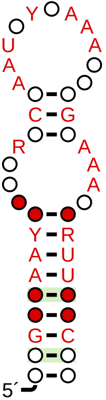

CRISPRCas_3_1

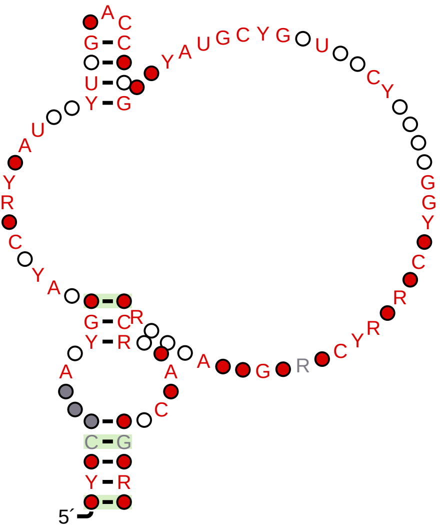

CRISPRCas_32_1

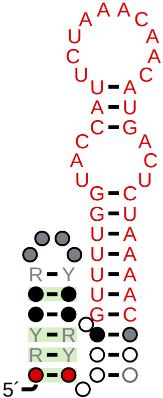

CRISPRCas_33_1

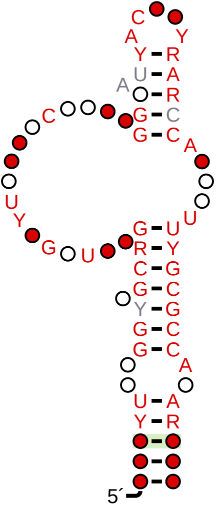

CRISPRCas_34_1

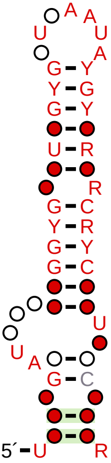

CRISPRCas_35_1

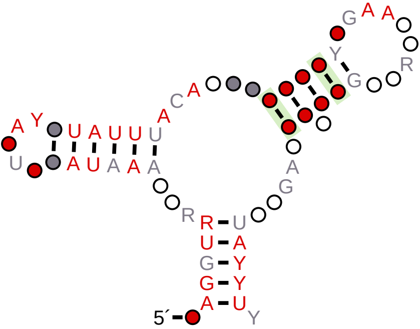

CRISPRCas_36_1

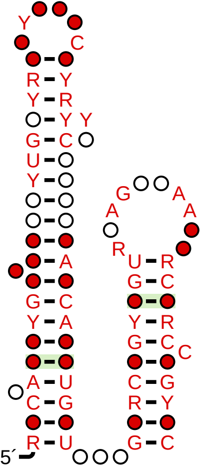

CRISPRCas_37_1

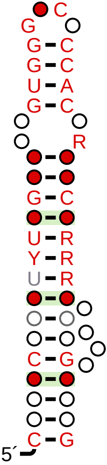

CRISPRCas_38_1

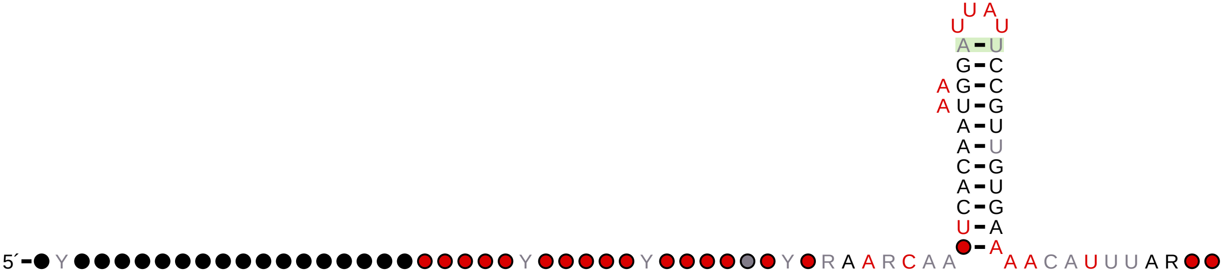

CRISPRCas_39_1

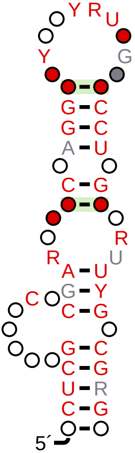

CRISPRCas_40_1

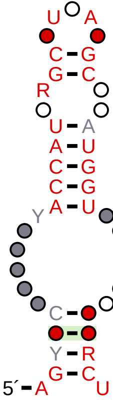

CRISPRCas_41_1

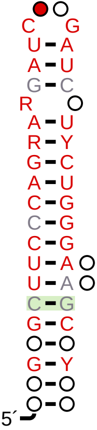

CRISPRCas_4_1

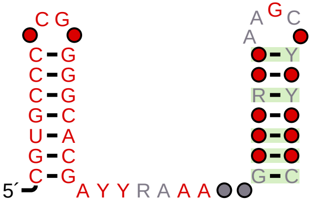

CRISPRCas_42_1

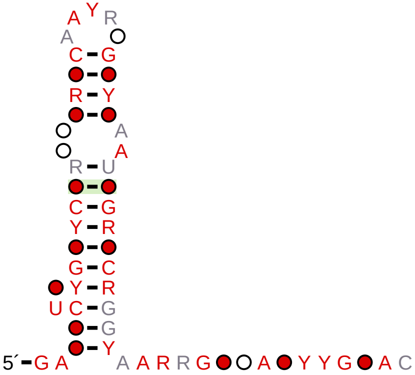

CRISPRCas_43_1

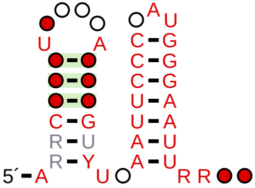

CRISPRCas_44_1

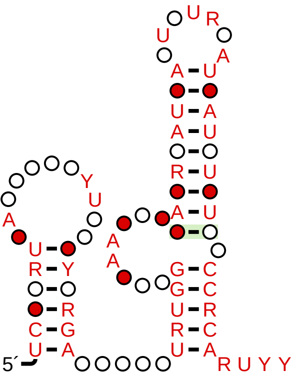

CRISPRCas_45_1

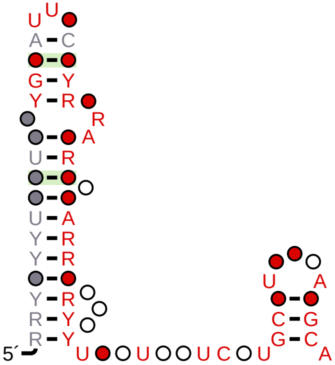

CRISPRCas_46_1

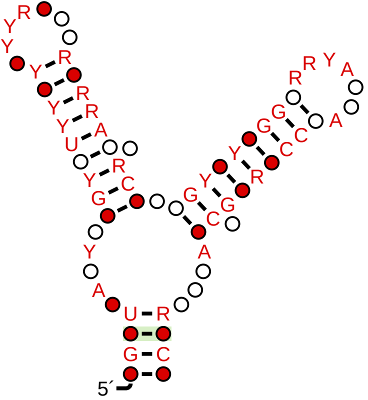

CRISPRCas_47_1

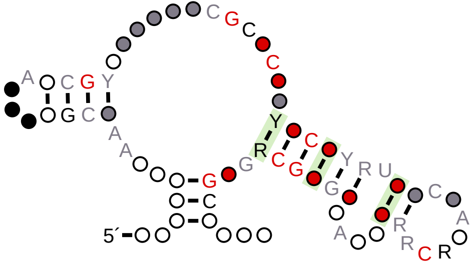

CRISPRCas_48_1

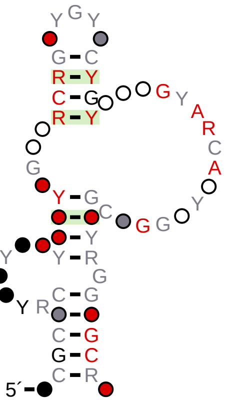

CRISPRCas_49_1

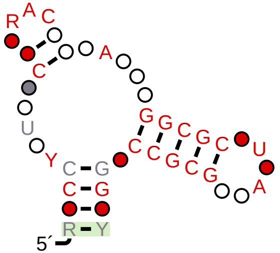

CRISPRCas_50_1

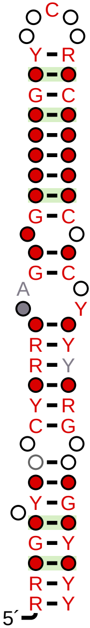

CRISPRCas_51_1

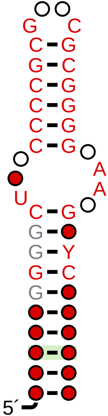

CRISPRCas_5_1

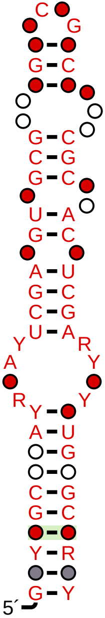

CRISPRCas_52_1

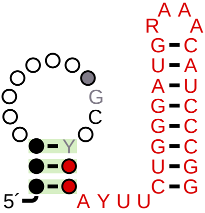

CRISPRCas_53_1

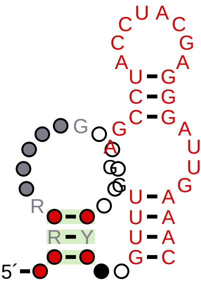

CRISPRCas_54_1

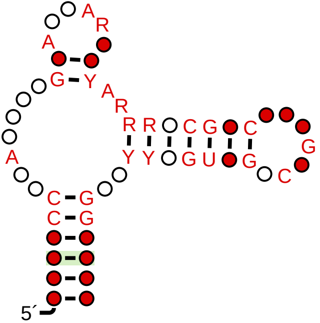

CRISPRCas_55_1

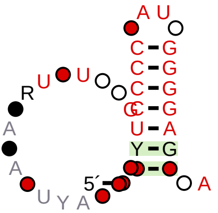

CRISPRCas_56_1

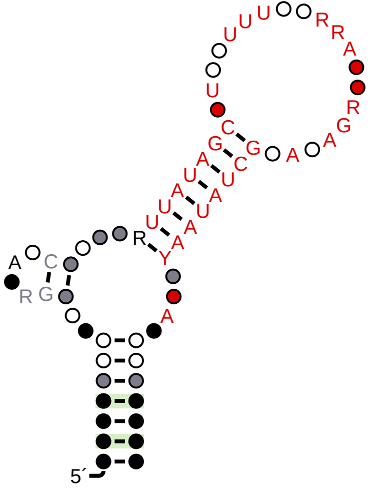

CRISPRCas_57_1

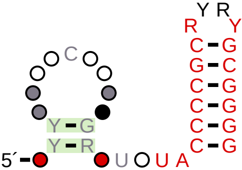

CRISPRCas_58_1

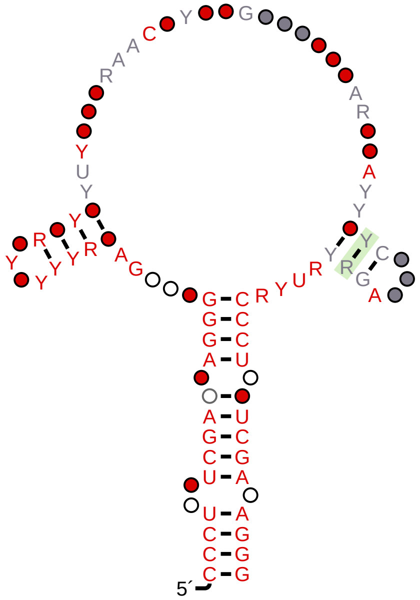

CRISPRCas_59_1

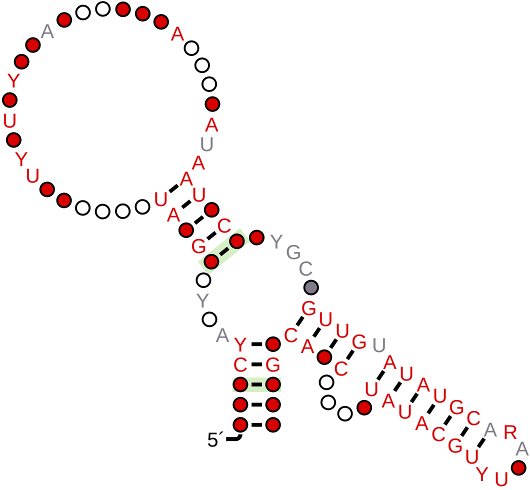

CRISPRCas_60_1

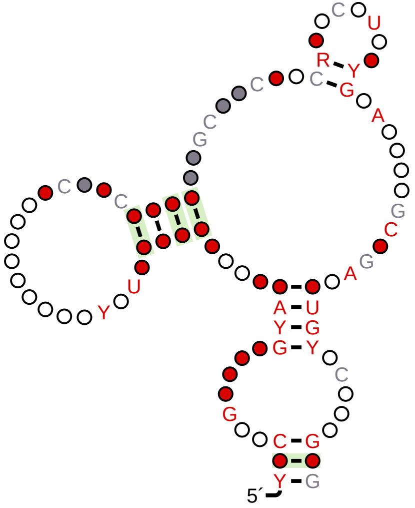

CRISPRCas_61_1

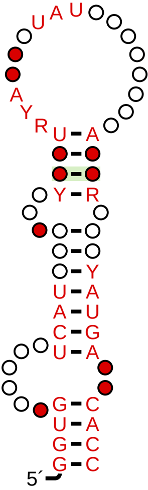

CRISPRCas_6_1

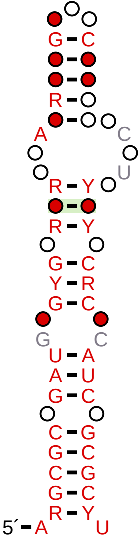

CRISPRCas_62_1

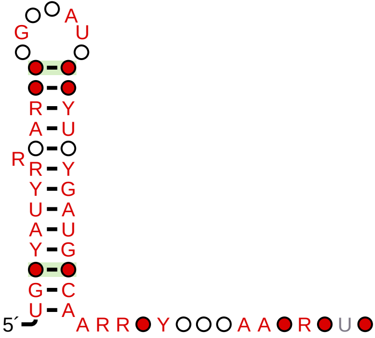

CRISPRCas_63_1

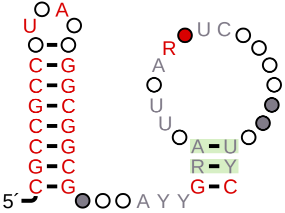

CRISPRCas_64_1

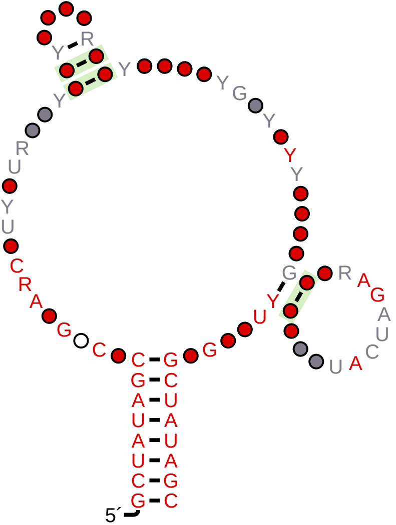

CRISPRCas_65_1

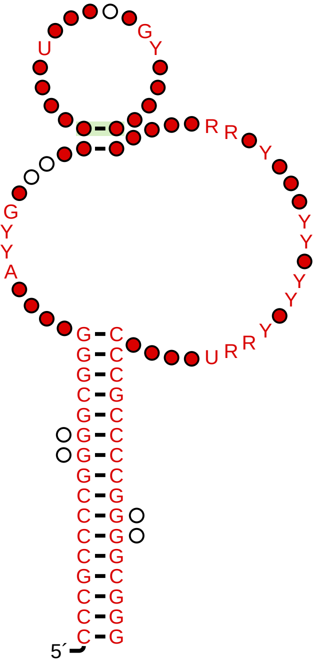

CRISPRCas_66_1

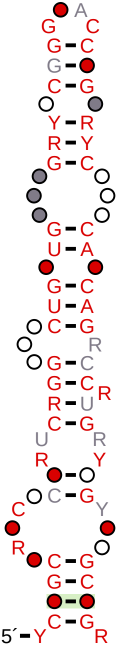

CRISPRCas_67_1

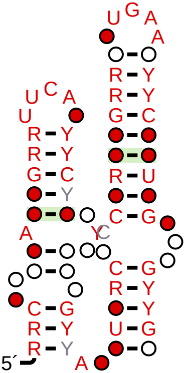

CRISPRCas_68_1

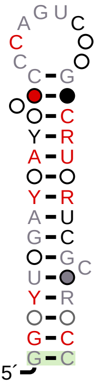

CRISPRCas_69_1

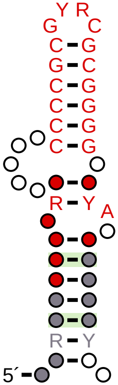

CRISPRCas_70_1

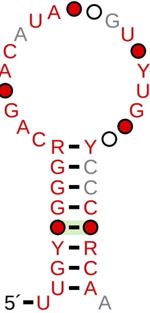

CRISPRCas_71_1

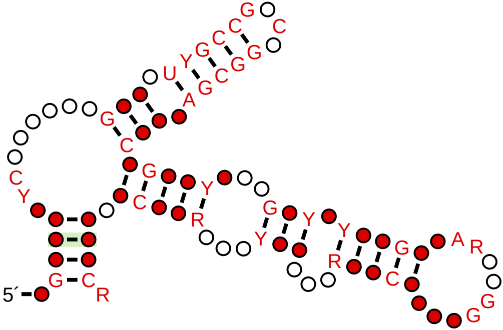

CRISPRCas_7_1

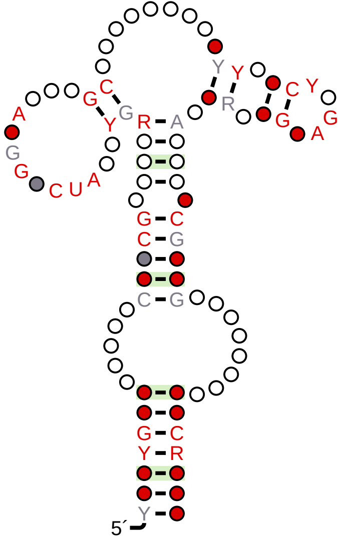

CRISPRCas_72_1

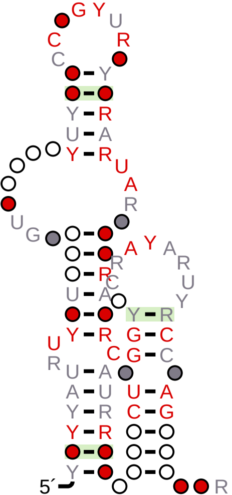

CRISPRCas_73_1

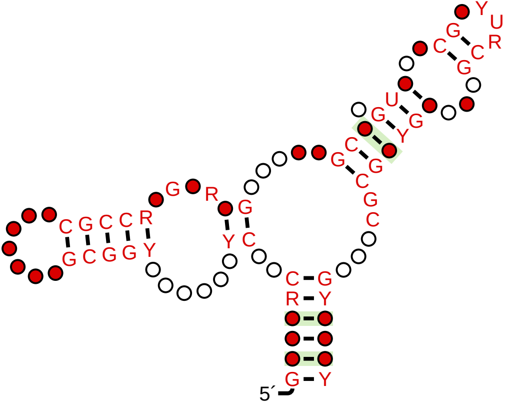

CRISPRCas_74_1

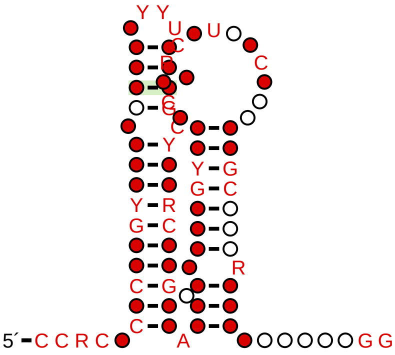

CRISPRCas_75_1

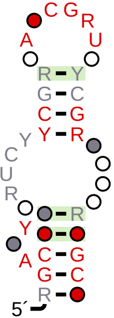

CRISPRCas_76_1

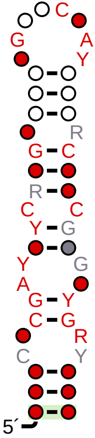

CRISPRCas_77_1

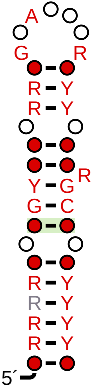

CRISPRCas_78_1

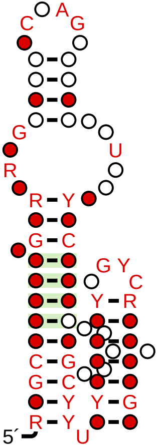

CRISPRCas_79_1

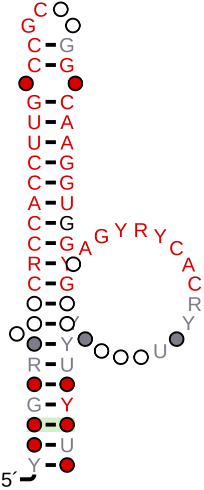

CRISPRCas_80_1

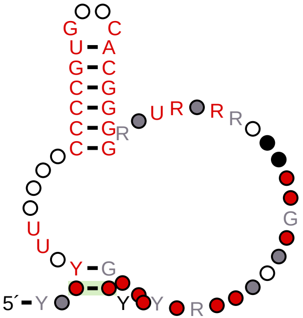

CRISPRCas_81_1

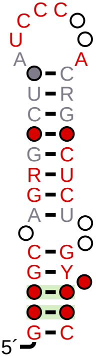

CRISPRCas_8_1

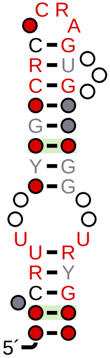

CRISPRCas_82_1

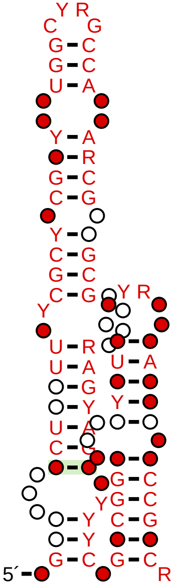

CRISPRCas_83_1

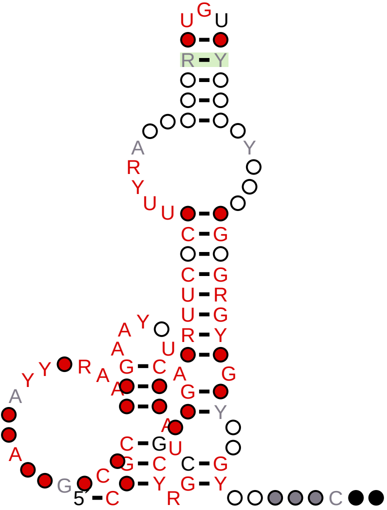

CRISPRCas_84_1

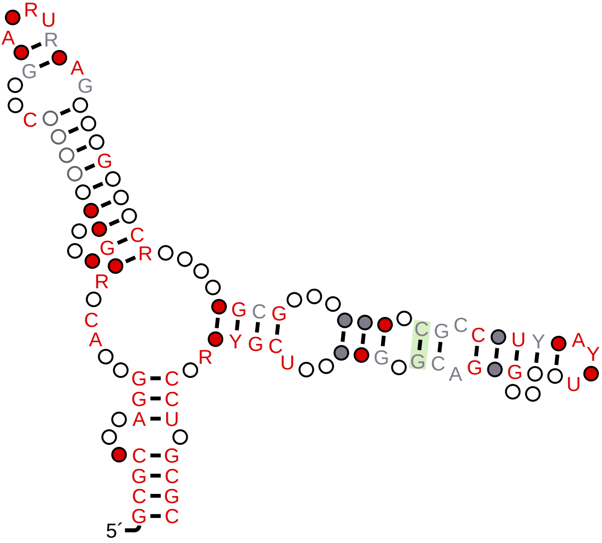

CRISPRCas_85_1

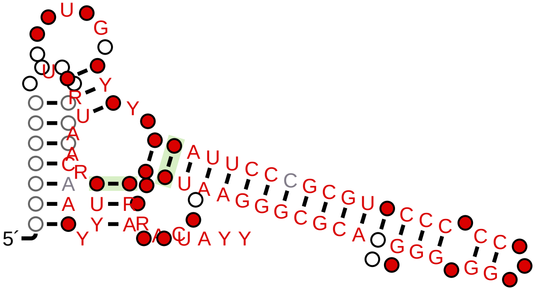

CRISPRCas_86_1

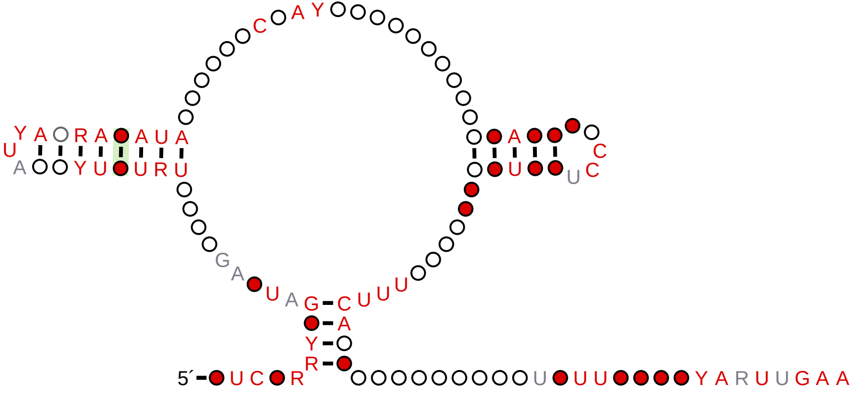

CRISPRCas_87_1

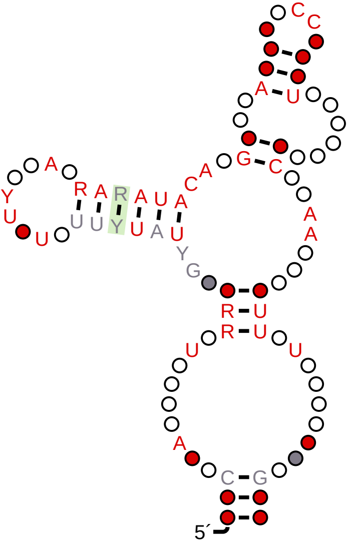

CRISPRCas_88_1

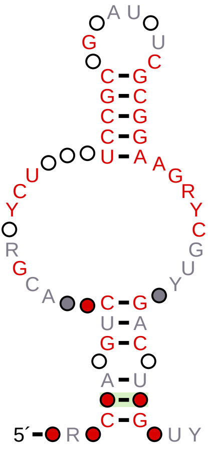

CRISPRCas_89_1

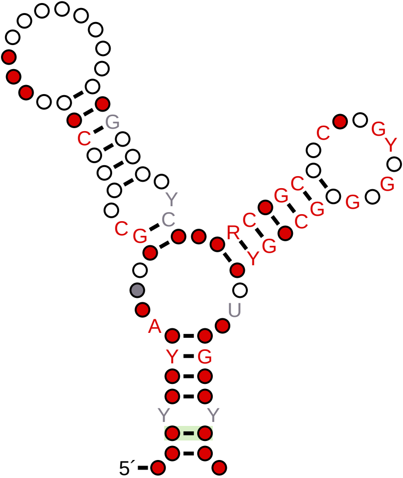

CRISPRCas_90_1

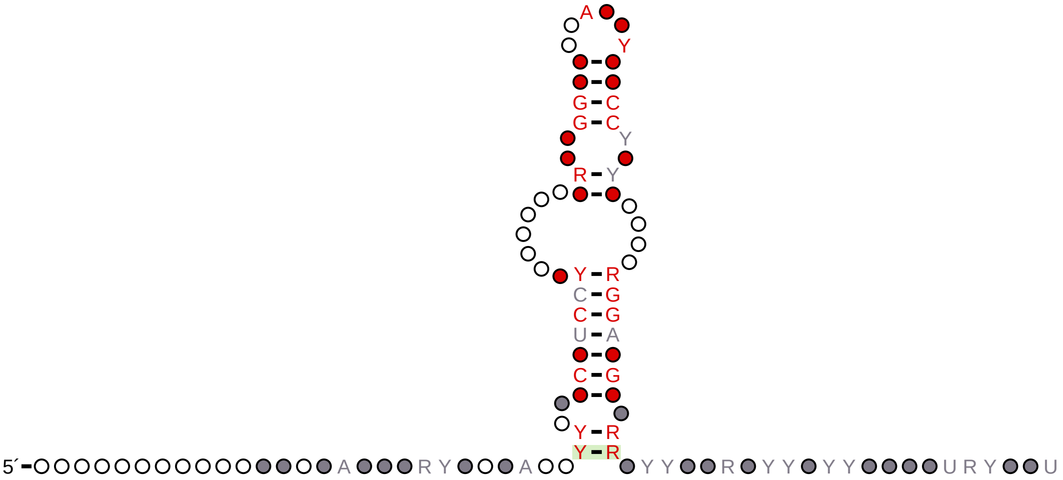

CRISPRCas_91_1

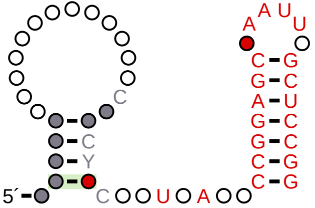

CRISPRCas_9_1

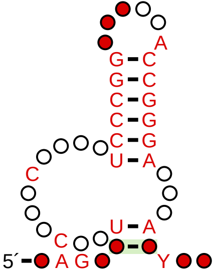

CRISPRCas_92_1

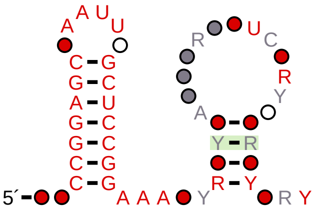

CRISPRCas_93_1

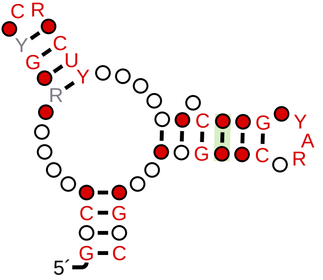

CRISPRCas_94_1

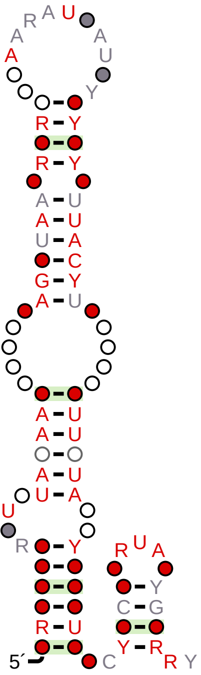

CRISPRCas_95_1

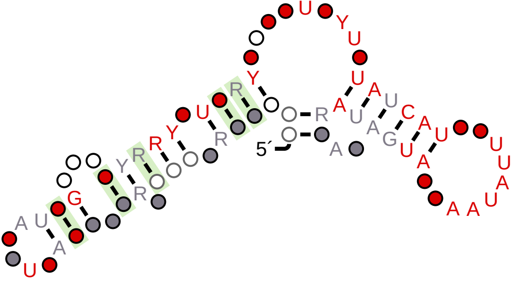

CRISPRCas_96_1

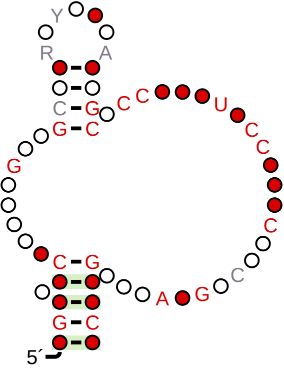

CRISPRCas_97_1

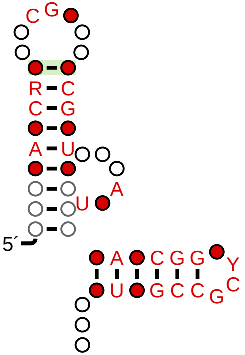

CRISPRCas_98_1

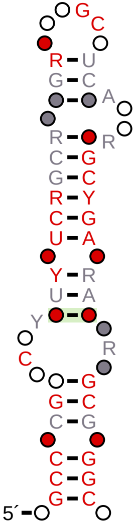

CRISPRCas_99_1

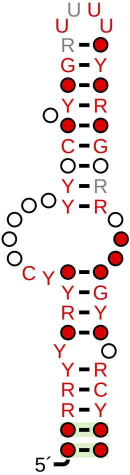

Supplement: Supplemental Material [file KRNB_A_2067714_SM8925.zip › FileS1_2.pdf]
