# Supplementary figures and images for: Identifying candidate structured RNAs in CRISPR operons
Source: RNA Biol. 2022 May 1;19(1):678–85. doi: 10.1080/15476286.2022.2067714 (PMC9067536; doi:10.1080/15476286.2022.2067714)

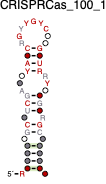

Supplement: Supplemental Material [file KRNB_A_2067714_SM8925.zip › FileS1_RNABiology.tiff]
